# Immunodominance of Lytic Cycle Antigens in Epstein-Barr Virus-Specific CD4+ T Cell Preparations for Therapy

**DOI:** 10.1371/journal.pone.0000583

**Published:** 2007-07-04

**Authors:** Dinesh Adhikary, Uta Behrends, Heike Boerschmann, Andrea Pfünder, Stefan Burdach, Andreas Moosmann, Klaus Witter, Georg W. Bornkamm, Josef Mautner

**Affiliations:** 1 Clinical Cooperation Group, Institute for Clinical and Molecular Biology, GSF-National Research Center for Environment and Health, Munich, Germany; 2 Children's Hospital, Hematology-Oncology, University of Technology, Munich, Germany; 3 Clinical Cooperation Group Molecular Oncology, Department of Gene Vectors, GSF-National Research Center for Environment and Health and Department of Otorhinolaryngology, Ludwig Maximilians University, Munich, Germany; 4 Laboratory of Immunogenetics, Ludwig Maximilians University, München, Germany; New York University School of Medicine, United States of America

## Abstract

**Background:**

Epstein-Barr virus (EBV) is associated with a number of human malignancies. EBV-positive post-transplant lymphoproliferative disease in solid organ and hematopoietic stem cell transplant recipients has been successfully treated by the adoptive transfer of polyclonal EBV-specific T cell lines containing CD4+ and CD8+ T cell components. Although patients receiving T cell preparations with a higher CD4+ T cell proportion show better clinical responses, the specificity of the infused CD4+ component has remained completely unknown.

**Methodology/Principal Findings:**

We generated LCL-stimulated T cell lines from 21 donors according to clinical protocols, and analyzed the antigen specificity of the CD4+ component in EBV-specific T cell preparations using a genetically engineered EBV mutant that is unable to enter the lytic cycle, and recombinantly expressed and purified EBV proteins. Surprisingly, CD4+ T cell lines from acutely and persistently EBV-infected donors consistently responded against EBV lytic cycle antigens and autoantigens, but barely against latent cycle antigens of EBV hitherto considered principal immunotherapeutic targets. Lytic cycle antigens were predominantly derived from structural proteins of the virus presented on MHC II via receptor-mediated uptake of released viral particles, but also included abundant infected cell proteins whose presentation involved intercellular protein transfer. Importantly, presentation of virion antigens was severely impaired by acyclovir treatment of stimulator cells, as currently performed in most clinical protocols.

**Conclusions/Significance:**

These results indicate that structural antigens of EBV are the immunodominant targets of CD4+ T cells in LCL-stimulated T cell preparations. These findings add to our understanding of the immune response against this human tumor-virus and have important implications for the improvement of immunotherapeutic strategies against EBV.

## Introduction

Epstein-Barr virus (EBV) is a ubiquitous human γ-herpesvirus implicated in the etiology of several tumors of lymphoid and epithelial origin [1]–[3]. Primary infection with EBV usually occurs early in life by parent-to-child oral transmission in an almost always asymptomatic fashion. Delayed primary infection in adolescence or adulthood may cause the syndrome of infectious mononucleosis (IM), a self-limiting lymphoproliferative disease [4]. After oral transmission, the virus replicates in the oropharynx, probably in the mucosal epithelium, from where it colonizes the host by latently infecting B cells. The reservoir of latently infected B cells can seed foci of virus replication at mucosal sites, and this reactivation of the virus and subsequent re-infection of B lymphocytes allows the virus to persist for life in the infected human host [5]. In B cells, EBV is able to establish different types of latency characterized by the expression of different sets of viral genes. During the primary phase of B cell infection, as well as in lymphoblastoid cell lines (LCL) generated by infection of B cells with EBV in vitro, the full range of eight antigenically distinct latent cycle proteins is expressed that drive the activation and proliferation of the infected cell [6], [7].

In vivo, outgrowth of latently infected growth-transformed B cells is curtailed by T cells. The importance of T cell-mediated immune responses in maintaining asymptomatic viral persistence is emphasized by the clinical observation that patients with T cell dysfunction are at risk of developing life-threatening EBV-associated lymphoproliferative disease [3]. In solid organ and hematopoietic stem cell transplant (HSCT) recipients, incidence of EBV-positive post-transplant lymphoproliferative disease (PTLD) correlates with the degree of the iatrogenically induced immunosuppression [3], [8]. Importantly, EBV-positive PTLD in HSCT recipients has been successfully treated by the adoptive transfer of EBV-specific T cell lines containing CD4+ and CD8+ components. These polyclonal lines are generated by repeated stimulation of peripheral blood T cells with irradiated autologous LCL in vitro [9]–[11]. The targets of LCL-stimulated CD8+ T cells have been studied in detail and display a marked hierarchy in immunodominance with epitopes derived from the EBNA3 family of proteins and immediate early as well as early lytic cycle proteins usually inducing the strongest responses across a range of HLA class I alleles [12]–[17]. The EBV-specific CD4+ T cell response is less well defined. In a recent phase II clinical trial, patients with PTLD showed better responses when the infused T cell preparations contained higher numbers of CD4+ T cells [18]. This study inferred an important role of CD4+ T cells in controlling EBV-driven lymphoproliferation, but the specificity of the CD4+ component in LCL-stimulated T cell preparations has remained completely unknown.

The proven safety and efficacy of adoptive T cell therapy for PTLD in HSCT recipients has provided an important proof of principle for this form of immunotherapy, but owing to the considerable technical requirements and financial implications of extensive in vitro T cell culture, adoptive T cell therapy still has a limited role in the management of virus-associated complications in transplant patients [19]. Nevertheless, despite a better understanding of PTLD pathogenesis and the development of early detection strategies such as serial measurement of EBV DNA load in peripheral blood samples, as well as the introduction of novel therapeutic agents such as antiviral drugs or monoclonal antibodies to CD20, adoptive T cell therapy is likely to remain an important therapeutic option for patients with tumors that fail to respond to antibody treatment, and to develop as a prophylactic option for patients who are identified as being at immediate risk of EBV-driven disease [8], [20].

Moreover, the successful treatment of PTLD in immunocompromised transplant recipients has encouraged the extension of these protocols to treat EBV-associated tumors developing in the presence of an apparently competent immune system, e.g. nasopharyngeal carcinoma (NPC) and Hodgkin's disease (HD). First clinical experience indicates that LCL-stimulated T cell lines may cause tumor regression in some cases but clinical responses are often partial and transient [21], most likely because of immune evasion strategies by tumor cells such as non-expression of the EBNA3 family of proteins, the immunodominant targets of the latent antigen-specific CD8+ T cell response [3], [8].

To increase clinical efficacy of the T cell preparations and to implement this treatment modality as a conventional therapeutic option, generic and more direct approaches for the generation of EBV-specific T cell lines enriched in disease-relevant specificities need to be developed. Prerequisite for the realization of these objectives is the knowledge of the relevant T cell antigens. Here, we studied the specificity of the CD4+ T cell component in LCL-stimulated T cell preparations.

## Materials and Methods

### Donors

Studies on material of human origin were approved by the ethics committees of the universities involved, and informed consent was obtained from all donors or their guardians. Blood samples from serologically confirmed cases of acute IM were obtained from the Children's Hospital, Munich University of Technology. Cord blood samples were provided by the University Hospital of the Ludwig Maximilians University, Munich. Mononuclear cells were isolated from blood samples by density gradient centrifugation on Ficoll-Paque (GE Healthcare). All donors were HLA-typed using PCR-based methods.

### Cell culture

LCL and mini-LCL were established by infection of primary B cells with B95.8 virus and the genetically engineered mini-EBV strain, respectively [22]. LCL, mini-LCL, the B95.8 marmoset cell line, and Burkitt's lymphoma cell lines were grown as suspension cultures in LCL media consisting of RPMI 1640, 10% fetal calf serum (FCS), 1% nonessential amino acids, 1mM sodium pyruvate, 2 mM L-glutamine, and 50 µg/ml gentamicin. HEK293T cells were cultured in DMEM medium supplemented with 10% FCS, 2 mM L-glutamine, and 50 µg/ml gentamicin. T cells were cultured in AIM-V media (Invitrogen) supplemented with 10% heat inactivated human serum, 2 mM L-glutamine, and 10mM HEPES. To avoid expansion of FCS-reactive T cells, all APCs used for T cell stimulation were grown in LCL media supplemented with 10% human serum instead of FCS. In some experiments, LCL treated with 200 µM acyclovir (Hexal) for at least for two weeks were used as T cell targets.

T cell lines were established by LCL or mini-LCL stimulation as in clinical protocols [9], [23]. After 4–8 passages, CD4+ cells were isolated from the T cell lines by positive or negative selection using α-CD4+ or α-CD8+ MicroBeads, LS-columns, and MidiMACS separator as recommended by the manufacturer (Miltenyi Biotec). T cell clones were generated by limiting dilution cloning in 96-well round-bottom plates.

Dendritic cells were differentiated from precursors in peripheral blood as described [24]. PHA blasts were generated by stimulating 10^6^/ml PBMC with 250 ng/ml PHA in T cell media supplemented with 50IU IL-2/ml.

### Phenotypic and functional analysis of T cells

For FACS analysis of T cells, FITC or PE-conjugated monoclonal antibodies against human CD4, CD8, and TCRα/β were used (all from Becton-Dickinson). TCR-Vβ usage by T cells was analyzed by RT-PCR and Southern blot. cDNA was synthesized from total RNA extracted from T cells and PCR performed using primers specific for the variable regions of the different human TCR-Vβ chains [25]. PCR products were separated in an agarose gel, blotted onto Hybond-N+ membrane (GE Healthcare) and hybridized with a TCR-Vβ chain constant region probe. IFN-γ ELISPOT assays and cytokine ELISAs were performed essentially as described [26]. Cytolytic activity of T cells was measured in europium ligand release assays [22].

### Preparation of concentrated EBV suspension

Cell free supernatant from B95.8 cells was filtered through a 0.8 µm filter and ultracentrifuged at 25,000× g for 3 hours in a SW28 rotor (Beckman Coulter). The supernatant was removed and the virus rich pellet resuspended in 1/20 volume of the original culture supernatant. The number of EBV genome equivalents (geq)/ml of this virus concentrate was determined by semi-quantitative real-time PCR using primers directed to the BALF5 gene [27].

### Expression and purification of EBV proteins

The following EBV proteins were selected: the latent proteins EBNA1, EBNA2, EBNA3A, EBNA3B, EBNA3C, EBNA-LP, LMP1, LMP2A; the immediate early lytic cycle proteins BZLF1 and BRLF1, the early lytic cycle proteins BALF1, BALF2, BALF3, BALF5, BaRF1, BARF1, BBLF2/BBLF3, BBLF4, BDLF4, BFLF2, BFRF1, BGLF3, BGLF5, BHRF1, BKRF3, BKRF4, BLLF3, BMLF1, BMRF1, BORF2, BRRF1, BVRF2, BXLF1, and the late lytic cycle proteins BALF4, BBRF1, BBRF2, BBRF3, BcLF1, BcRF1, BCRF1, BDLF1, BDLF3, BFRF3, BGLF1, BGLF2, BILF2, BKRF2, BLLF1, BLRF1, BLRF2, BNRF1, BOLF1, BORF1, BSLF1, BSRF1, BXLF2, BXRF1, BZLF2. The cDNAs coding for latent cycle proteins were kindly provided by Dr. W. Hammerschmidt (GSF, Munich), or cloned from latently infected cells. The lytic cycle genes were amplified by PCR from B95.8 virus DNA and all genes cloned into the CMV promoter/enhancer driven mammalian expression vector pCMV-EHis, tagging the EBV genes at their 3′ end with sequences coding for the epitope recognized by the monoclonal α-EBNA1 antibody 1H4, and a His-tag consisting of six consecutive histidines.

For recombinant protein expression, the plasmids were transiently transfected into HEK293T cells using the calcium phosphate transfection method [28]. The cells were harvested 48 to 60 hours after transfection and lysed in urea lysis buffer (8 M Urea, 0.1 M NaH_2_PO_4_, 0.01 M Tris, 0.05% Tween 20, 20 mM imidazole; pH 8.0). Following centrifugation (5,000× g/15 min) to pellet insoluble debris, the His-tagged proteins were purified using Nickel-NTA agarose beads according to the guidelines of the manufacturer (Qiagen). The protein eluate was dialysed against PBS, the concentration determined using Bradford reagent (BioRad), and the solutions brought to a concentration of 50 µg/ml. The proteins were separated by SDS-PAGE, and identity and purity analyzed by Coomassie staining and by Western blot using the 1H4 monoclonal antibody (kindly provided by Dr. E. Kremmer, GSF, Munich) and the ECL plus detection system (GE Healthcare). For antigen identification, APC were incubated overnight with 1 µg/ml recombinant protein, excess protein removed by washing, and probed with T cells.

## Results

### Generation of CD4+ T cell lines using LCL as stimulators

**Figure 1:**
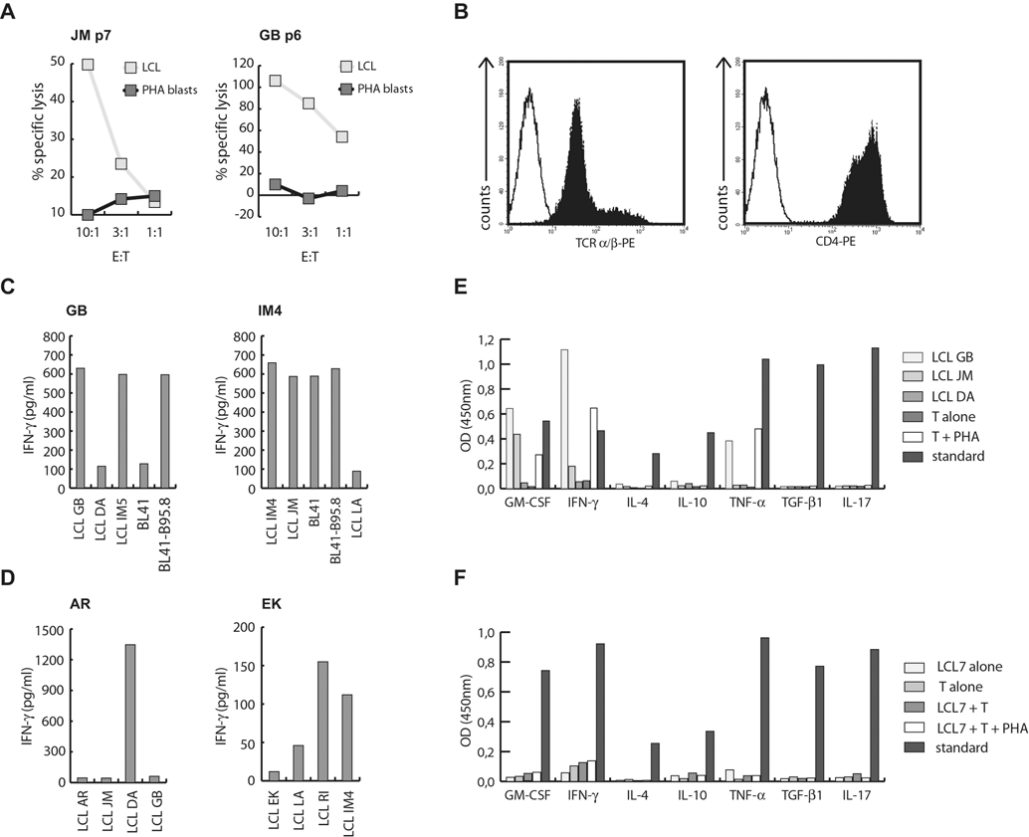
Generation and characterization of LCL-stimulated CD4+ T cell lines. T cell lines established from EBV-positive donors by LCL stimulation lysed autologous LCL but not PHA blasts after 4–8 passages at different effector-to-target (E:T) ratios. (B) FACS analysis of CD4+ cell lines established from LCL-stimulated bulk T cell lines by magnetic sorting demonstrated that >95% of the cells were TCRα/β+ and CD4+. (C) As demonstrated for donor GB, all T_H_ cell lines established from healthy virus carriers responded against autologous and MHCII-matched allogeneic LCL, as well as MHCII-matched EBV-positive (BL41-B95.8) but not EBV-negative (BL41) Burkitt's lymphoma cell lines. T_H_ cell lines from IM patients showed similar responses against autologous LCL, but as exemplified by the T cell line from IM4, some of these lines also recognized EBV-negative BL cell lines. (D) LCL-stimulated T_H_ cell lines from EBV-negative donors showed minimal if any responses against autologous LCL, but vigorous responses against some allogeneic targets. (E) EBV-reactive T_H_ cell lines secreted GM-CSF, IFN-γ, and TNF-α, but not IL-4, IL-10, IL-17, or TGF-β1 in response to stimulation with autologous (GB) or MHCII-matched allogeneic (JM) LCL, or non-specific activation by PHA. The MHC-mismatched LCL DA served as negative control. The following standards were included: GM-CSF: 1,900 pg/ml; IFN-γ: pg/ml; IL-4: 250 pg/ml; IL-10: 2,100 pg/ml; TNF-α: 2,900 pg/ml; TGF-β1: 1,450 pg/ml; IL-17: 1,700 pg/ml. (F) The T cell line IM7 displayed a novel “non-responder” phenotype. This T cell line proliferated in response to stimulation with autologous LCL and IL-2, but failed to secrete any of the indicated cytokines even after stimulation with autologous LCL plus PHA.

Using autologous LCL as stimulators, T cell lines were established from mononuclear cells of umbilical or peripheral blood of 21 individuals; five cord blood donors, eight patients with IM, and eight healthy adult volunteers of whom seven were EBV-seropositive and one EBV-seronegative. As described for LCL-stimulated T cell lines prepared for clinical applications [10], [29], all T cell lines established from EBV-seropositive donors lysed autologous LCL but not PHA blasts after 4–8 rounds of stimulation (Fig. 1A). To study the CD4+ T cell components, all T cell lines were enriched for CD4+ T helper (T_H_) cells by selecting CD4+ cells. FACS analysis of the sorted T cell lines verified that all lines contained more than 95% TCRα/β+ and CD4+ cells, and this phenotype was maintained over extended periods of in vitro culture (Fig. 1B). When tested for target-specific cytokine secretion, T cell lines from all EBV-positive donors recognized autologous and allogeneic LCLs in an MHCII-restricted fashion. LCL-stimulated T_H_ cell lines from healthy virus carriers also recognized MHCII-matched EBV-positive but not or only weakly EBV-negative target cells (Fig. 1C left), indicating that these lines were specific for EBV antigens. Of the eight T cell lines derived from IM patients, three displayed EBV-specificity by these criteria whereas five T cell lines responded similarly against EBV-negative and EBV-positive target cells (Fig. 1C right), implying that these T cells recognized self-antigen(s), or were specific for viral antigens and coincidentally cross-reacted against alloantigens.

The five T cell lines derived from cord blood, and the T cell line from the EBV-seronegative healthy adult barely recognized autologous LCL (Fig. 1D). Because some of these cell lines responded vigorously against MHCII-mismatched target cells, T cell unresponsiveness was unlikely to account for this low reactivity. These results demonstrated that EBV-specific T_H_ cell memory is efficiently reactivated by LCL stimulation, and excluded that de novo priming of EBV-specific T_H_ cell responses occurs under these in vitro conditions.

Upon target cell recognition all LCL-reactive T cell lines predominantly secreted Th1 cytokines (Fig. 1E), except for the T cell line derived from IM7. This T cell line proliferated upon stimulation with autologous LCL and IL-2 and was maintained in culture for more than 50 passages, but failed to secrete any of the cytokines tested in response to autologous or allogeneic targets or when stimulated with PHA (Fig. 1F).

### Latent cycle antigens of EBV are not the principal targets of LCL-stimulated EBV-specific CD4+ T cells

**Figure 2:**
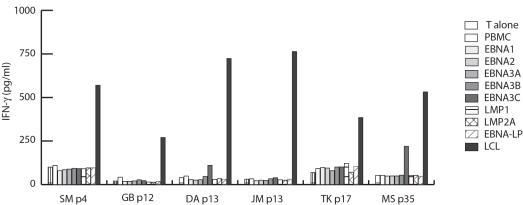
Latent cycle antigens of EBV are not the principal targets of LCL-stimulated T_H_ cells. EBV-specific T_H_ cell lines from different donors at different passages were tested for recognition of autologous PBMC pulsed separately with the eight antigenically distinct latent cycle proteins of the virus. Except for the T cell lines from donors DA and MS, which showed weak responses against EBNA3C, neither early nor late passage T_H_ cell lines responded against EBV latent cycle proteins.

The exclusive recognition of EBV-positive but not EBV-negative targets by the T cell lines established from all healthy virus carriers and three IM patients suggested that these T cells were directed against latent cycle proteins of EBV. To define the T_H_ cell antigens molecularly, all eight antigenically distinct latent cycle proteins of EBV were recombinantly expressed and the purified proteins pulsed on autologous PBMC, which were subsequently used as targets for the EBV-specific T cells. Efficient presentation of peptides derived from latent cycle proteins on MHC II was verified in control experiments using CD4+ T cell clones specific for five of the latent antigens of EBV (data not shown). Surprisingly, except for two T cell lines showing weak responses against EBNA3C, none of the EBV-specific T cell lines recognized any of the latent cycle antigens of EBV (Fig. 2). Because CD4+ T cells specific for latent antigens of EBV have been isolated from peripheral blood of healthy virus carriers by different groups [26], [30]–[33], and T cells specific for five different latent cycle antigens have been isolated previously from three of the healthy EBV-seropositive donors included in this study (data not shown), the absence of latent cycle antigen-specific T_H_ cell responses was unlikely to account for these negative results. Since the precursor frequency of such T cells in peripheral blood is generally low [33], up to 50 restimulations were performed to facilitate the expansion of such rare T cell specificities to detectable levels. No reactivity against any of the latent cycle antigens was detected in these late passage T cell lines. Even the weak responses against EBNA3C were no longer detected (data not shown), indicating that LCL-stimulated T_H_ cell lines either target non-latent cycle antigens of EBV or cellular antigens induced by EBV.

### Lytic cycle antigens are the immunodominant targets of EBV-specific CD4+ T cell lines

**Figure 3:**
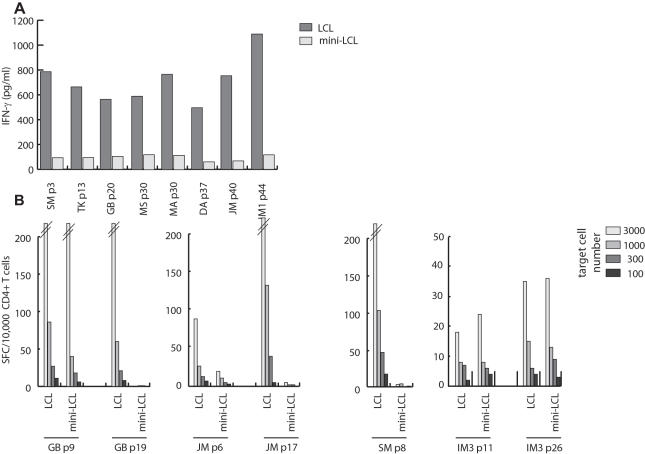
EBV-reactive T_H_ cell lines recognize autologous LCL but not mini-LCL. LCL-stimulated T_H_ cell lines showing EBV reactivity were tested for recognition of autologous LCL and mini-LCL established by infection of B cells with an EBV mutant unable to enter the lytic cycle. After three to twenty passages, mini-LCL reactivity of all T_H_ cell lines had dropped to background levels while responses against LCL were maintained even after extended periods of in vitro culture. (B) Responses against LCL and mini-LCL of different passage T_H_ cell lines were assessed by IFN-γ ELISPOT. With the exception of the T cell line from SM, early passage T_H_ cell lines from healthy virus carriers recognized LCL and mini-LCL, but responses against mini-LCL disappeared with further rounds of stimulation. By contrast, early and late passage T cell lines from IM3, which had failed to show EBV-reactivity in earlier experiments, responded similarly against both types of target cells. SFC, spot forming cells.

To address whether LCL-stimulated T cells recognize lytic cycle antigens of EBV, mini-LCL incapable of expressing lytic cycle proteins were established by infecting B cells with a genetically engineered mutant strain of EBV and used as T cell targets [22], [34]. While early passage T cell lines responded similarly against LCL and mini-LCL, responses against mini-LCL decreased to background levels with further rounds of stimulation in all T cell lines that had shown EBV specificity in previous experiments, suggesting that the late passage T cell lines recognized lytic cycle antigens or cellular genes induced by EBV infection (Fig. 3A). To assess the T cell responses against mini-LCL versus LCL in more detail, IFN-γ ELISPOT assays were performed. Loss of mini-LCL reactivity became apparent after three to twenty passages depending on the cell line analyzed, but eventually all lines established from healthy virus carriers reacted against LCL but not or minimally against mini-LCL (Fig. 3B). By contrast, the four T cell lines established from patients with IM that had shown EBV-independent LCL-reactivity in previous experiments continued to respond against both types of target cells (Fig. 3B). The recognition of EBV-negative target cells in earlier experiments suggested that these lines targeted cellular antigens.

### Immunodominance of autoantigens over viral latent cycle antigens

The weak and transient responses against EBNA3C detected in two of the T cell lines established from healthy virus carriers implied that T cells specific for latent cycle antigens expanded under these in vitro culture condition, albeit less efficiently than lytic cycle antigen-specific T_H_ cells. To assess whether latent cycle antigen-specific T cells are a subdominant component of the LCL-stimulated T_H_ cell response, CD4+ PBMC from the donors DA and JM were repeatedly stimulated with autologous mini-LCL. These donors were chosen because T_H_ cell lines and clones specific for EBV latent cycle antigens had been established previously from their peripheral blood, predicating the presence of such T_H_ cell specificities in the peripheral memory compartment (data not shown). The resulting T cell lines responded similarly against autologous mini-LCL and LCL. Surprisingly, except for weak responses against EBNA3C in donor DA, these lines failed to recognize autologous PBMC or DC pulsed with any of the latent cycle antigens of EBV even after more than 25 passages, demonstrating that these T cell were not specific for latent antigens of EBV, but targeted cellular antigen(s) (Fig. S1).

### Immunodominance of virion antigens

**Figure 4:**
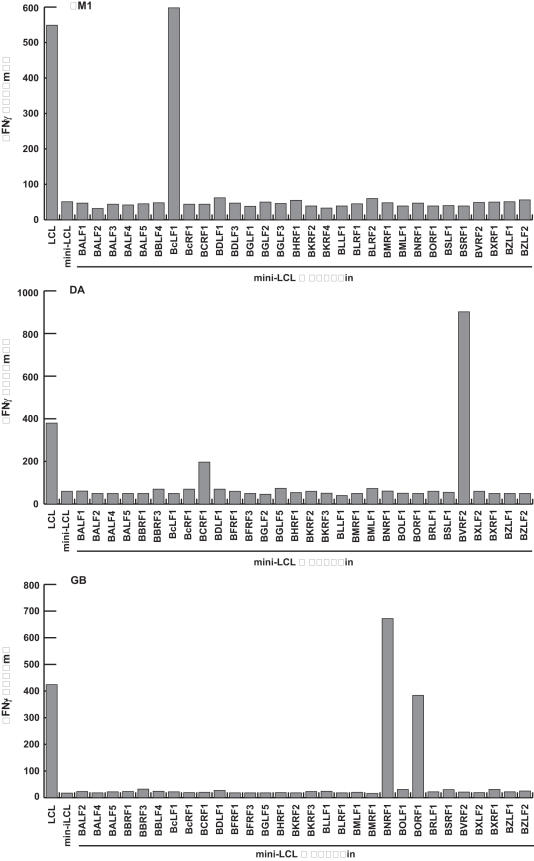
EBV-reactive T_H_ cell lines target lytic cycle antigens of the virus. The antigens recognized by T_H_ cell lines that responded against LCL but had lost mini-LCL reactivity were identified using mini-LCL pulsed with recombinant EBV proteins. Responses of three representative CD4+ T cell lines (IM1, DA, and GB) against 30 different lytic cycle proteins are shown.

To investigate if the LCL but not mini-LCL-reactive T_H_ cell lines recognized lytic cycle antigens, we cloned 50 of the more than 80 different lytic cycle genes of EBV including the immediate early antigens *BZLF1* and *BRLF1*, 23 early antigens, and 25 late antigens. Mini-LCL were pulsed with the recombinantly expressed and purified lytic cycle proteins and subsequently probed with the T cell lines once mini-LCL reactivity had subsided. All T cell lines recognized at least one of the lytic cycle antigens tested (Fig. 4). Except for the lytic cycle proteins BMRF1, BCRF1, and BALF2, all of the antigens identified were derived from virion proteins (Table 1). Moreover, all lines targeted at least one virion antigen. With the notable exception of the tegument protein BNRF1, which was recognized by six of the ten T cell lines, diverse sets of antigens were recognized by the different T cell lines, suggesting that the immunodominant antigens of the LCL-stimulated T_H_ cell populations are derived from structural proteins of EBV (Table 1).

**Table 1:** Summary of the antigens recognized by EBV-reactive T_H_ cell lines

Donor	dominant antigens	subdominant antigens
IM1	BcLF1	BFRF3, BXLF2
IM2	BXLF2	BDLF1, BNRF1
IM5	BNRF1	BALF2
GB	BNRF1	BXRF1, BORF1, BDLF1, BBRF3
JM	BALF2	BDLF1, BXRF1, BALF4
DA	BVRF2	BNRF1, BCRF1, BORF1, EBNA3C
MS	BALF4	EBNA3C
SM	BALF2, BNRF1	BMRF1
MA	BMRF1, BNRF1	Nd
TK	BORF1	Nd

The antigens recognized by the EBV-reactive T_H_ cell lines were identified by using PBMC or mini-LCL pulsed with single latent or lytic cycle proteins of EBV as targets. Responses against dominant antigens were maintained up to fifty restimulations, while responses against subdominant antigens were detected at early passages of the T_H_ cell lines only. Nd, not determined.

### Late passage LCL-stimulated T_H_ cell lines are still oligoclonal

Although late passage T cell lines usually responded against a single lytic cycle antigen, these experiments left unresolved whether these lines were still oligoclonal and contained additional specificities that remained undetected in these experiments e.g. lytic cycle antigens that had not been included in this study. To address this issue, two sets of experiments were performed. First, selected T cell lines were cloned by limiting dilution and analyzed for antigen specificity by assessing recognition of LCL, mini-LCL, and mini-LCL pulsed with proteins identified as targets of the parental T cell line. This analysis revealed that only a portion of the single cell outgrowths was of expected specificity. For example, the clones obtained from the T cell line MA passage p17 could be subdivided into four groups: (i) those that recognized BNRF1 or BMRF1, the previously identified targets of the parental T cell line, (ii) those that recognized LCL but neither mini-LCL alone nor mini-LCL pulsed with the recombinant BNRF1 or BMRF1, (iii) those that responded against LCL as well as mini-LCL and (iv) those that secreted neither GM-CSF nor IFN-γ upon co-culture with the target cells (Fig. S2). Thus, in addition to T cells recognizing the antigens identified in the parental cell line, this line contained T cells specific for additional and still unidentified lytic cycle antigen(s), autoreactive T cells, and T cells with a similar non-responder phenotype as noted before with the T cell line from IM7. Since the antigens recognized by the last three types of T cells were unknown, it remained unclear whether the clones within the same group recognized one or several antigens. T cell clones established from late passage T cell lines usually lacked autoantigen-specificity, but still recognized more than one antigen (data not shown). In a second set of experiments, T_H_ cell lines were analyzed for T cell receptor Vβ chain (TCR-Vβ) expression. T_H_ cell lines stimulated more than 40 times were still positive for more than one TCR-Vβ chain (Fig. S3), demonstrating that LCL-stimulated T_H_ cell lines from EBV-positive individuals remain oligoclonal even beyond a year and a half in culture.

### Presentation of lytic cycle antigens on MHC II

**Figure 5:**
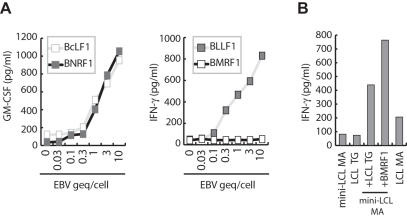
Lytic antigens are transferred between cells by virions and released proteins. CD4+ T cells specific for BLLF1, BMRF1, BcLF1 or BNRF1 were tested for recognition of mini-LCL pulsed with purified viral particles. Whereas BcLF1, BLLF1, and BNRF1-specific T cells responded against mini-LCL pulsed with less than 1 genome equivalent (geq) of the virus/cell, BMRF1-specific T cells failed to recognize mini-LCL pulsed with much higher doses of virus. (B) To detect transfer of antigen between cells, BMRF1-specific T cells were tested for recognition of mini-LCL, MHC-mismatched LCL, and the mix of these two lines. While neither line alone was recognized by the T cells, 24 hours of co-culture sensitized the cell mix for recognition.

Given the low percentage of usually less than 1% of cells in an LCL culture that spontaneously become permissive for lytic replication, it was surprising to find that lytic cycle proteins of EBV are the immunodominant targets recognized by LCL-stimulated T cell lines. To investigate if structural proteins of EBV are presented via the receptor-mediated presentation pathway recently described for EBV glycoproteins [22], mini-LCL were pulsed with viral particles and tested for recognition by virion-specific T cells. Because purified EBV particles also contain low amounts of the non-structural early lytic cycle proteins BALF2 and BMRF1 [35], T cells specific for BMRF1 were included in this analysis. Mini-LCL pulsed with less than 1 genome equivalent (geq)/cell were recognized by all virion-specific, but not by BMRF1-specific T cells, demonstrating that virion antigens are efficiently presented on MHC II and that the number of BMRF1 molecules in virions is probably insufficient for T cell detection (Fig. 5A). To address whether the presentation of BMRF1 involved intercellular protein transfer as recently described for latent cycle proteins [36] and the lytic cycle protein BHRF1 [37], autologous mini-LCL were co-cultured with MHC-mismatched LCL for 24 hours. T cell recognition of the cell mixture, but neither component alone, indicated that antigen released from cells undergoing lysis is transferred to neighboring cells (Fig. 5B). Since BMRF1 and BALF2 are highly abundant infected cell proteins, such a scenario may also explain why LCL-stimulated T cell lines frequently targeted these antigens.

### Acyclovir treatment of LCL severely impairs late lytic cycle antigen presentation on MHC II

**Figure 6:**
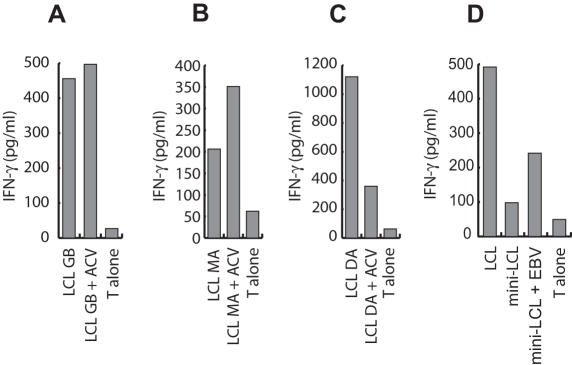
Presentation of virion antigens is impaired in acyclovir-treated LCL. LCL either left untreated or treated with acyclovir for two weeks were used as targets for BMRF1 (A), autoantigen (B), or BNRF1-specific T cells (C). Acyclovir treatment neither affected presentation of the autoantigen nor the EBV early lytic cycle antigen BMRF1, but severely reduced the presentation of the virion antigen BNRF1. (D) T cell lines generated by repeated stimulation of peripheral blood CD4+ cells with acyclovir-treated LCL recognized LCL and mini-LCL that had been pulsed with purified EBV particles, suggesting that late lytic cycle antigen-specific T cells still expand under these stimulation conditions.

To preclude transfer of infectious virus into patients, T cell lines for clinical use are usually prepared by stimulation with acyclovir-treated LCL [38], [39]. Because acyclovir limits virus production by interfering with late lytic cycle protein expression, we compared T cell recognition of LCL cultured in the presence or absence of acyclovir for two weeks. While acyclovir treatment did not affect recognition of LCL by autoantigen and BMRF1-specific T cells, recognition by BNRF1-specific T cells was severely impaired (Fig. 6A). Similar results were obtained in co-culture experiments of acyclovir-treated allogeneic LCL and autologous mini-LCL (data not shown) demonstrating that treatment of LCL with this drug selectively diminishes the presentation of late lytic cycle antigens. To assess whether acyclovir-treated LCL still released enough virus to reactivate late lytic cycle antigen-specific T cell memory, CD4+ cells from peripheral blood were stimulated with acyclovir-treated LCL as for clinical applications [38], [39]. Recognition of LCL and virus-pulsed mini-LCL, but not untreated mini-LCL, by these T cells indicated that acyclovir-treated LCL are still able to expand virion antigen-specific T cells, albeit to a much lesser extent than untreated LCL (Fig. 6B, and data not shown).

## Discussion

The reconstitution of EBV-specific immunity in HSCT recipients by the adoptive transfer of polyclonal virus-specific T cell lines has provided an important proof of principle for immunotherapy of EBV-associated tumors, and for cancer immunotherapy in general [40]–[43]. Given the significant burden of EBV-associated tumors worldwide, important future goals of this adoptive T cell therapy are the introduction into mainstream clinical practice and the extension to EBV-associated tumor entities other than PTLD [21], [40]. In a prelude to facilitate and expedite the preparation of T cell lines enriched in disease-relevant T cell effectors, the specificity of LCL-stimulated CD4+ T_H_ cell preparations was analyzed and followed over time. Early passage T cell lines from all EBV-positive, but not virus-naïve donors, responded against lytic cycle and autoantigens. With further rounds of stimulation, all T cell lines from healthy virus carriers responded predominantly against lytic cycle antigens, while late passage T cell lines from IM patients were often dominated by autoreactive rather than virus-specific T cells. Surprisingly, latent cycle antigens of EBV were barely targeted by LCL-stimulated and even mini-LCL-stimulated T cell lines. This was unexpected because all latent cycle proteins are expressed in LCL, and CD4+ T cells specific for latent antigens have been detected in the peripheral blood of EBV-seropositive donors, including three of the healthy virus carriers analyzed in this study. These results indicate that latent cycle antigen-specific T_H_ cells are either a minor component of the LCL-reactive T_H_ cell memory compartment, or peptides derived from latent proteins are inefficiently presented on MHC II.

The second unexpected finding of this study was that LCL-stimulated T_H_ cell lines contained a high proportion of autoreactive T cells, which either displayed a typical Th1, or a novel “non-responder” phenotype. The latter T cells were detected among T cell clones established from most of the lines and dominated the late passage T cell line from IM7, which makes them a relevant component of the LCL-stimulated T_H_ cell population. The definition of T cell effector functions is essential for a more detailed characterization of this unusual T cell subset. Autoreactive T cells of Th1 type were detected in EBV-infected individuals only, and these specificities dominated the LCL-stimulated T cell cultures from several IM patients, suggesting a link between acute EBV infection and the induction of autoreactive T_H_ cell responses. Of note, autoreactive T cells have recently been described as component of the CD4+ T cell response that suppresses the outgrowth of LCL from newly EBV-infected B cells in regression assays [44]. Thus, autoreactive T cells could play a protective role against EBV infection, albeit at the expense of damaging normal tissues. Autoimmunity, however, has not been observed in HSCT recipients treated with LCL-stimulated T cell lines, implying that these T cells are suppressed in vivo. Nevertheless, several autoimmune diseases including multiple sclerosis [45], systemic lupus erythematosus [46], and rheumatoid arthritis [47] have been linked to EBV infection. For elucidating whether these T_H_ cells contribute to the pathogenesis of autoimmune diseases, it will be important to identify the antigens recognized by these T cells.

The most important finding of this study was the unexpected immunodominance of lytic cycle antigens. Most of these antigens were derived from late lytic gene products that belong to the group of structural proteins of EBV. This immunodominance may be a reflection of the efficient presentation of virion antigens on MHC II following receptor-mediated uptake and processing in the lytic compartment [22], [48], and may explain why LCL-stimulated T_H_ cell lines target such a broad set of virion antigens. Besides structural proteins, the lytic cycle antigens BCRF1, BMRF1, and BALF2 also elicited T_H_ cell responses, and responses to BALF2 prevailed over structural antigens in one of the donors. The presentation of BMRF1 involved intercellular antigen transfer, probably by release of protein from lytically infected cells and uptake as exogenous protein by neighboring cells as described previously [36], [37]. Why these but not other lytic cycle proteins like BZLF1 [22] are efficiently transferred between cells is not known, but might reflect quantitative differences in protein expression levels. By which pathways BALF2 and BCRF1 are presented is currently not known.

The identification of the immunodominant and subdominant antigens of LCL-stimulated T_H_ cell preparations has several clinical implications. First, in order to minimize residual infectious viral particles within adoptively transferred T cells, most currently applied clinical protocols use acyclovir to suppress virus production in stimulator LCL [38], [39]. Inhibition of late lytic cycle protein expression by this drug, however, diminishes virion antigen presentation and may lead to a preponderance of autoantigen-specific T_H_ cell responses. Thus, generating T_H_ cell lines enriched in late lytic cycle antigen-specific effectors may necessitate the modification of current stimulation protocols. Second, the failure of most of the autoreactive T cells to recognize PBMC and DC suggests that using these cells as stimulators may reduce the autoreactive component in T_H_ cell preparations. Evidence in support of this proposition has been obtained in recent experiments showing that significantly fewer rounds of stimulation are required to generate virion-specific T_H_ cell lines when using virus-pulsed PBMC rather than LCL as APC (data not shown). Such modified stimulation protocols would further expedite the preparation of EBV-specific T_H_ cell lines by obviating the lengthy procedure of establishing LCL. Third, the inefficient expansion of latent cycle antigen-specific T_H_ cells by LCL stimulation, most importantly CD4+ T cells specific for EBNA1 which is expressed in all EBV-associated malignancies, implies that incorporating these effectors into the T cell preparations may further improve their clinical efficacy. Finally, the identification of the immunodominant targets of the EBV-specific T_H_ cell response provides insight into the role of this T cell subset in the control of EBV infection. By recognizing and eliminating newly infected cells, virion-specific T_H_ cells limit the spreading of infection and keep the pool of latently infected B cells small [22], [49]. Interestingly, LCL-stimulated CD8+ T cells are predominantly directed against latent cycle as well as immediate early and early lytic cycle antigens [14]–[17], [50]. Targeting mostly non-overlapping sets of viral proteins and different phases of the virus' life cycle implies that CD4+ and CD8+ T cells complement each other in establishing protective immunity against EBV.

Although most EBV-associated tumors express MHC II, the low number of tumor cells undergoing lytic replication in vivo challenges the concept of an analogous role of virion-specific CD4+ T cells in tumor control. However, the efficient transfer of virion antigens to bystander cells by receptor-mediated uptake of released viral particles, which results in T_H_ cell recognition of target cells incubated with less than one viral particle per cell, suggests that only few tumor cells undergoing lysis may sensitize a large proportion of tumor cells for T_H_ cell recognition. Moreover, radio/chemotherapy of EBV-positive tumors in vivo is associated with the induction of lytic replication in a significant portion of tumor cells, and more selective compounds for reactivating EBV from latency are currently evaluated [51], [52]. The combination of lytic cycle induction strategies with T cell therapy may even be more effective than either approach alone and may further improve the clinical effectiveness of this form of immunotherapy and the long term survival of patients.

## Supporting Information

Figure S1 Latent cycle proteins of EBV are not the dominant targets of mini-LCL stimulated T_H_ cell lines. Mini-LCL-stimulated CD4+ T cell lines from donor DA and JM were tested for recognition of latent cycle proteins of EBV by using PBMC or DC preincubated for 24 hours with recombinant latent cycle proteins as targets in T cell cytokine secretion assays. The T cells from donor JM failed to show above-background response against any of the latent cycle proteins whereas the T cells from donor DA showed only minimal response against EBNA3C.
(0.91 MB EPS)

Figure S2 Late passage LCL-stimulated CD4+ T cell lines are oligoclonal. Single cell clones of the LCL-stimulated T cell line from donor MA at passage 17 were tested for recognition of LCL, mini-LCL, and mini-LCL pulsed with BNRF1 or BMRF1, the antigens recognized by the parental T cell line. Of 16 clones analyzed, 5 were BMRF1-specific, 1 was BNRF1-specific, 4 recognized LCL but not mini-LCL, 2 showed significant responses against LCL and mini-LCL, and 4 failed to secrete IFN-γ in response to any of the target cells.
(0.76 MB EPS)

Figure S3 T cell receptor Vβ chain analysis of late passage T cell lines. T cell receptor Vβ chain expression of T_H_ cell lines, that had shown lytic cycle antigen specificity, was analyzed by RT-PCR using Vβ chain specific primers and subsequent Southern blot hybridization of the PCR products. Even late passage T cell lines still expressed more than one Vβ chain, demonstrating that the T cell lines were still oligoclonal. The T_H_ cell line from donor SM, that had already lost mini-LCL reactivity after four stimulations, still expressed multiple Vβ chains, indicating that many different lytic cycle antigen-specific T_H_ cells may exist in healthy virus carriers.
(5.54 MB EPS)
